# Effect of Structure of Polymers Grafted from Graphene Oxide on the Compatibility of Particles with a Silicone-Based Environment and the Stimuli-Responsive Capabilities of Their Composites

**DOI:** 10.3390/nano10030591

**Published:** 2020-03-24

**Authors:** Monika Zygo, Miroslav Mrlik, Marketa Ilcikova, Martina Hrabalikova, Josef Osicka, Martin Cvek, Michal Sedlacik, Barbora Hanulikova, Lukas Munster, David Skoda, Pavel Urbánek, Joanna Pietrasik, Jaroslav Mosnáček

**Affiliations:** 1 Department of Chemistry, Lodz University of Technology, Institute of Polymer and Dye Technology, Stefanowskiego 12/16, 90 924 Lodz, Polandupolmail@savba.sk (M.I.); 2 Centre of Polymer Systems, University Institute, Tomas Bata University in Zlin, Trida T. Bati 5678, 760 01 Zlin, Czech Republic; hrabalikova@cps.utb.cz (M.H.); osicka@utb.cz (J.O.); cvek@utb.cz (M.C.); msedlacik@utb.cz (M.S.); hanulikova@cps.utb.cz (B.H.); munster@utb.cz (L.M.); dskoda@cps.utb.cz (D.S.); urbanek@cps.utb.cz (P.U.); 3 Polymer Institute, Slovak Academy of Sciences, Dubravska cesta 9, 845 41 Bratislava 45, Slovakia; 4 Department of Polymer Engineering, Faculty of Technology, Tomas Bata University in Zlin, Vavreckova 275, CZ-76272 Zlin, Czech Republic; 5 Centre for Advanced Material Application, Slovak Academy of Sciences, Dubravska cesta 9, 845 11 Bratislava, Slovakia

**Keywords:** graphene oxide, SI-ATRP, smart composites, compatibility, grafting, conductivity

## Abstract

This study reports the utilization of controlled radical polymerization as a tool for controlling the stimuli-responsive capabilities of graphene oxide (GO) based hybrid systems. Various polymer brushes with controlled molecular weight and narrow molecular weight distribution were grafted from the GO surface by surface-initiated atom transfer radical polymerization (SI-ATRP). The modification of GO with poly(*n*-butyl methacrylate) (PBMA), poly(glycidyl methacrylate) (PGMA), poly(trimethylsilyloxyethyl methacrylate) (PHEMATMS) and poly(methyl methacrylate) (PMMA) was confirmed by thermogravimetric analysis (TGA) coupled with online Fourier transform infrared spectroscopy (FTIR), transmission electron microscopy (TEM) and X-ray photoelectron spectroscopy (XPS). Various grafting densities of GO-based materials were investigated, and conductivity was elucidated using a four-point probe method. Raman shift and XPS were used to confirm the reduction of surface properties of the GO particles during SI-ATRP. The contact angle measurements indicated the changes in the compatibility of GOs with silicone oil, depending on the structure of the grafted polymer chains. The compatibility of the GOs with poly(dimethylsiloxane) was also investigated using steady shear rheology. The tunability of the electrorheological, as well as the photo-actuation capability, was investigated. It was shown that in addition to the modification of conductivity, the dipole moment of the pendant groups of the grafted polymer chains also plays an important role in the electrorheological (ER) performance. The compatibility of the particles with the polymer matrix, and thus proper particles dispersibility, is the most important factor for the photo-actuation efficiency. The plasticizing effect of the GO-polymer hybrid filler also has a crucial impact on the matrix stiffness and thus the ability to reversibly respond to the external light stimulation.

## 1. Introduction

Particle surface chemistry is a crucial factor that influences the performance of two-phase systems, where the particles create a discontinues phase dispersed in a continues one. The surface properties determine the affinity of the discontinuous phase/material to the matrix environment and thus affect the interface performance. The effects can be observed through the estimation of the degree of dispersion and homogeneity of distribution of the particles, control of morphology, and selective particle localization in immiscible blends or block copolymer matrices [1,2]. The surface chemistry, in fact, has a strong impact on the final physical properties of the blend such as thermal, mechanical or electrical properties of the systems [3]. Even more interesting are the effects in smart systems, such as liquid or solid stimuli-responsive materials. 

Photomechanical actuators belong to the class of stimuli-responsive materials as they can provide mechanical motion as a response to external light stimuli. Such materials find utilization in engineering applications such as robots [4], pens for molecular printing [5], smart curtains, light-driven motors [6], or treating of amphiphilic nano-objects by light-induced charge separation [7]. Photo-actuators are based on hybrid particles with certain specific characteristic features. They contain hard and soft phases, where the hard phase provides the shape stability, and the soft phase enables the shape changes [8]. The properties of the polymer matrix network also play an important role, since it determines the stability of the materials during actuation. Systems based on both chemical and physical cross-linking have been studied. The latter includes thermoplastic elastomers (TPE) such as styrene-isoprene-styrene [9], poly(methyl methacrylate)-*b*-poly(butyl acrylate)-*b*-poly(methyl methacrylate) [10,11], ethylene-vinyl acetate [12,13], ethylene terpolymer containing epoxy, butyl and ethylene groups [14]. The main advantages of TPEs is their easy and reversible processing, and cost-effectivity when commercially available materials are used. The risk of the utilization of TPEs is connected with the thermal stability of the physical cross-linking, which can be disturbed during applications at high temperatures. Additionally, the utilization of fillers may even hinder the formation of the physical cross-linking network, such as for example, the dispersion of carbon nanotubes (CNT) in polystyrene-*b*-polyisoprene-*b*-polystyrene (SIS) due to strong π-π interactions of styrene groups with the conjugated structure of added CNT [15]. However, suitable surface modification can provide a solution of this issue, to some extent. Nonetheless, the utilization of the cross-linked systems overcomes this disadvantage. The systems based on liquid crystals have been studied intensively [9,16,17,18]. The actuation response has been enhanced by the addition of azo-moieties, that are however highly toxic, into polymer structures [19,20]. Compared to previously mentioned matrix materials, poly(dimethyl siloxane) is easy to process, is nontoxic and provides the ability for sufficient photo-actuation [14,21,22,23]. 

Carbon-based fillers have been studied intensively since they provide a photo-actuation response to infrared light [9] and it has been revealed that the alignment of CNTs enhances the responsive efficiency [24]. Polymer chains tend to align along the CNT surface and thus CNT facilitates polymer chains orientation in the direction of actuation and enhance the actuation stroke. Nevertheless, the shape of the particles plays a more significant role. Graphene-based particles have been determined to be better candidates due to their two-dimensional shape, as well as large surface area [25]. Graphene particles are of particular interest since they provide high electrical conductivity, excellent mechanical properties and high thermal conductivity, that facilitate heat transfer within the sample [26], and can also improve the photo-actuation capability [27]. However, the dispersion and distribution of neat graphene nanoparticles in a polymer is rather problematic due to the strong π-π interaction between the graphene sheets [28]. Graphene surface properties can be altered to overcome this drawback. Oxidation of the surface, thereby introducing oxygen-containing functional groups poses an efficient strategy for surface modification [29]. Depending on the degree of oxidation, the GO loses electrical conductivity since the conjugated structure is disturbed [30], however the modified particles possess better compatibility with the surrounding polymer matrix [31]. In addition, the functional groups also enable further post-modification reactions to obtain surface chemistry compatible with any targeted polymer matrix [32,33]. SI-ATRP is the most versatile modification method. It allows control over the molecular chain length, grafting density, topology [34,35], and as was demonstrated recently, the electrical conductivity of GO [36,37].

Similar to research in photomechanical actuators, research in rheological fluids have also turned attention to graphene-based systems [38,39]. Graphene oxide particles have been examined as a promising dispersed phase due to several specific properties, i.e., they can be prepared in a suitable range of electrical conductivity, they are lightweight, and therefore do not significantly form a sediment, as similar to hybrid particles of titanate nanotubes and polypyrrole [40]. Moreover, they can be prepared in a cost-effective way. In our previous research using SI-ATRP for preparation of hybrid particles, we proved that graphene oxide polymer hybrids with tailored polymer chain architecture, possessing good affinity to silicone oil, and with controlled electrical conductivity to fit parameters needed for ER applications can be efficiently prepared. The shear stress of materials obtained by our discussed methods reached 5 to 95 Pa at a low concentration of particles (10 wt. %) [41,42], which are promising values for real-life applications. 

We limited our study to silicone-based systems, i.e. silicone oil as a continuous liquid phase and silicone elastomer as a continuous phase of a solid-like system. Silicone oil is used as a carrier liquid in electrorheological suspensions, while silicone elastomer has been proved as a suitable matrix for the preparation of mechanical actuators [43,44]. Our previous research showed that SI-ATRP is a powerful tool for tailoring the ER performance of ER fluids based on graphene oxide and silicone oil [45]. Similarly, the studies of poly(dimethyl silioxane) (PDMS) filled with graphene oxide polymer hybrids (GO-*g*-polymer) photo-actuators also revealed that through surfaced modification we were able to improve the affinity GO-*g*-polymer particles to a PDMS matrix. SI-ATRP enables one to finely tune the properties of the chemical cross-linking network in terms of decreasing the activation energy of glass transition (systems possess enhanced flexibility of the PDMS network) that finally improves the ability for photo-actuation [21,22,27].

Herein, we extend the research to examine GO grafted with four different compositions of polymer chains to modulate GO surface chemistry and provide the complex comparison of the surface modification with respect to affinity to two types of silicon-based environment. Thus, the GO hybrids were tested both in silicon oil, a liquid commonly used in ER fluids, and in PDMS, a solid like matrix, with potential photomechanical actuation capability. The polymer brushes on the GO surface were synthesized by SI-ATRP. Comparable structures with respect to polymer chain length and grafting density of the polymers chains on the GO surface were prepared. The influence of the GO hybrids structure on the final properties of the stimuli-responsive systems were elucidated.

## 2. Materials and Methods 

### 2.1. Materials

Graphite (powder, synthetic, average particle size lower than 20 μm) was used and was purchased from (Sigma Aldrich, Poznan, Poland). Sulfuric acid (H_2_SO_4_, 95–98%), sodium nitrate (NaNO_3_, ACS reagent, 99%), potassium permanganate (KMnO_4_, 97%) and hydrogen peroxide (H_2_O_2_, ACS reagent, 29.0–32.0 wt % H_2_O_2_ basis) from (Sigma Aldrich, Poznan, Poland) were used as the chemical reagents for the exfoliation conditions required to form the GO sheets. Glycidyl methacrylate (98%), methyl methacrylate (98%), *n*-butyl methacrylate (98%), 2-(trimethylsilyloxy)ethyl methacrylate (HEMATMS, 99%), anisole (98%), copper bromide (CuBr, 97%), *N,N,N′,N″,N″*-pentamethyldiethylenetriamine (PMDETA, 98%), ethyl 2-bromoisobutyrate (EBiB, 98%), 2-bromoisobutyryl bromide (BiBB, 98%) were acquired from (Sigma Aldric, MO, USA) and used without further purification. Tetrahydrofuran (THF) (POCH, Gliwice, Poland) and triethylamine (Et_3_N) 98% from (Fluka, Buchs, Switzerland) were dehydrated over sodium before the use. Silicone elastomer (PDMS) Sylgard 184 and silicone oil (SO) Lukosiol M200 were purchased from (Dow, Midland, MI, USA) and (Lukoil, Prague, Czech Republic), respectively. Acetone p.a., dimethylformamide p.a. and diethyl ether p.a. (LachNer, Neratovice, Czech Republic) were used as received. Hydrochloric acid (HCl, ACS reagent, 37%) was supplied by (Sigma Aldrich, Poznan, Poland) [46].

### 2.2. Methods

#### 2.2.1. Preparation of GO from Graphite

The surfaces of graphite particles were oxidized using a modified Hummers method. Instead of oxidation for two hours as is discussed in the original paper [46] in this case the oxidation was performed over 8 h to generate the desired carboxyl, hydroxyl and epoxy functional groups. In this procedure, 5 g of graphite was stirred with 100 mL of H_2_SO_4_. The mixture was cooled down to 5 °C in an ice/water bath and stirred for 8 h. In the next step, 300 mL of deionized water was added dropwise to the dispersion, while the temperature was kept below 40 °C. Then, 40 mL of concentrated H_2_O_2_ was added resulting in a color change. The color was brilliant brown, indicating oxidation of the graphite surface. Finally, the product was separated using a high-speed centrifuge (Sorvall LYNX 4000, Thermo Scientific, Waltham, MA, USA) at 10,000 rpm at 25 °C for 20 min. The collected product was cleaned by re-dispersion in 0.1 M HCl and re-separated under centrifugation. This step was repeated several times using deionized water until the pH of the contacting water reached a value of 7. Finally, GO particles were freeze-dried to remove residual water. 

#### 2.2.2. Modification of GO Surface with ATRP Initiator (GO-I)

A 1000 mL three-neck round-bottom flask with 10 g of GO was subjected to three freeze-thaw-pump cycles. Freshly distilled THF (300 mL) was injected into the flask and then the flask and contents were sonicated for 4 min. Et_3_N (21.7 mL, 27 mmol) was added to the reaction flask followed by the dropwise addition of the ATRP initiator, 2-bromoisobutyryl bromide (16.5 mL, 27 mmol). The reaction mixture was left under stirring overnight at RT and then the reaction was heated to reflux at 80 °C for one-hour. The resulting GO-I product, was purified using a poly(tetrafluoroethylene) (PTFE) 0.2 μm membrane. The solid collected on the membrane was washed with acetone (100 mL). GO -I was then dispersed in DMF (200 mL) and sonicated for 30 s. Then it was filtered and washed with acetone. The above-mentioned procedure of purification was repeated three times. Finally, the GO-I was washed with diethyl ether and left in a vacuum oven at 60 °C for 12 h.

#### 2.2.3. General Procedure for SI-ATRP from GO-I Surface

1.5 g of GO-I and a magnetic stirrer was placed into a 100 mL Schlenk flask. The content of the reactor was degassed using three freeze-thaw-pump cycles. Anisole (30 mL) was purged with argon and added to the Schlenk flask. PMDETA and the sacrificial initiator, EBiB were injected into the reaction mixture which was sonicated in an ultrasonic bath for 1 min. 30 mL of monomer previously deoxygenated with argon, was added to the Schlenk flask followed by four freeze-pump-thaw cycles. During the final cycle CuBr was added to the frozen contents of the Schlenk flask under argon flow. The precise amount of the individual components in reaction mixture can be calculated from the Table 1. Then, the polymerization was conducted at 70 °C and stopped after 2 h by removing Schlenk flask from the bath and opening the flask to air. The reaction mixture was dissolved in another 15 mL of acetone and purified according to the procedure described in 2.2.2. Molar ratios of all reagents are presented in Table 1 together with the monomer conversion calculated based on the ^1^H NMR spectra as well as average number molar mass (*M*_n_) and molar masses dispersity (*Đ*) determined by gel permeation chromatography for polymer prepared by initiation from the added sacrificial initiator.

#### 2.2.4. Methods for GO and GO-*g*-Polymer Hybrids Characterization

A gel permeation chromatograph, PL-GPC220 (Agilent, Tokyo, Japan), was used for the determination of *M*_n_ and *Đ* of the polymer chains initiated from the sacrificial initiator (flow rate = 1.0 mL min^−1^, solvent: THF). Anisole was used as an internal standard and polystyrene as a standard. 

Monomer conversions were checked by proton nuclear magnetic resonance (^1^H NMR) using a 400 MHz VNMRS Varian NMR spectrometer equipped with 5 mm 1H-19F/15N-31P PFG AutoX DB NB probe. Samples were measured in deuterated chloroform at 25 °C. 

The morphology of neat GO and GO after surface modification was studied utilizing a Philips CM12 transmission electron microscope (Philips, Amsterdam, The Netherlands) with a resolution of 0.35 nm. A dispersion of particles in chloroform (1 mg mL^−1^) was sonicated in an ultrasonic bath for 3 min, and then drop cast onto a carbon-plated copper grid.

The number of GO layers before and after modification was estimated using atomic force microscopy (AFM, Dimension ICON) manufactured by Bruker company (Billerica, MA, USA). A dispersion of particles in chloroform (1 mg mL^−1^) was sonicated in an ultrasonic bath for 3 min, and then drop cast onto a freshly cleaned silicon wafer substrate. The observation was performed immediately after the solvent evaporated.

The surface area of GO and GO-*g*-polymer hybrids powders was characterized using a volumetric sorption analyzer, Belsorp mini-II (Microtracbel, Tokyo, Japan).

X-ray diffraction (XRD MiniFlex600, Rigaku, Japan) was used to determine the distance between the layers in both GO and GO-*g*-polymer hybrids powders. The XRD diffractometer was equipped with Co Kα source (*λ* of 0.17903 nm, 40 kV and 20 mA) and scans were collected in the range 2*θ* between 5° and 90°.

The Fourier transform infrared (FTIR) spectra of neat GO and GO-*g*-polymer hybrids powders were collected using a Nicolet iS10 (Thermo Scientific, Waltham, MA, USA). FTIR equipment was coupled with the TGA (Labsys evo, Setaram Instrumentation, France), while the components formed during the decomposition over the range from 50 to 650 °C at a heating rate 5 °C min^−1^ were continuously analyzed by IR. 

X-ray photoelectron spectroscopy (XPS) measurements of GO and GO-*g*-polymer hybrids powders were carried out using a TFA XPS instrument (Physical Electronics, Feldkirchen, Germany). The base pressure in the analytical chamber was approximately 6 × 10^−8^ Pa. Monochromatic Al Kα1,2 radiation at 1486.6 eV was used for sample excitation. Photoelectrons entered a hemispherical analyzer at an angle of 45° with respect to the normal of the sample surface. XPS survey-scan spectra were taken at pass energy of 187.9 eV, using an energy step of 0.5 eV, whereas high-resolution spectra of carbon C 1s were measured at pass energy of 29.35 eV using an energy step of 0.13 eV. MultiPak v8.1c software (Version 8.1c, Ulvac-Phi Inc., Kanagawa, Japan, 2006) from Physical Electronics was used for spectra analysis. All spectra were referenced to the main carbon atom C 1s peak, which was assigned a value of 284.8 eV. C1s spectra were fitted using a symmetrical Gauss-Lorentz function and Shirley-type background subtraction was applied to decompose spectral peaks. 

The Raman shift was investigated utilizing a Nicolet DXR spectrometer (Thermo Scientfic, Madison, WI, USA) resolution of 2 cm^−1^, excitation wavelength of 532 nm, 3 scans. The laser power on the surface was set to 1 mW and the integration time was 30 s. The samples were in the form of the powder.

For conductivity measurements, powders of synthesized neat GO and modified GO-PGMA samples were pressed into pellets of 13 mm diameter and 0.3–0.4 mm thickness at 400 MPa. For the density determination, the prepared GO-based pellets were weighted in the air and then immersed in decane by means of Analytical Balances Sartorius R160P (Sartorius AG, Goettingen, Germany). The conductivity of GO samples was measured via the van der Pauw method using Keithley 6517B electrometer (Keithley, Solon, OH, USA) at room temperature. 

Contact angle measurements (CA) of prepared GO-based pellets were performed through the static sessile drop method utilizing a Surface Energy Evaluation device with a CCD camera (Advex Instruments, Brno, Czech Republic). The drop of silicone oil Lukosiol M200 used for investigation had a volume of 5 μL and the values presented are the average number with standard deviation from 5 individual measurements. 

#### 2.2.5. Methods for Electrorheological Suspensions Characterization

Suspensions for sedimentation stability measurements, as well as for rheological measurements, were prepared by mixing neat GO or polymer modified GO particles with the corresponding amount of Lukosiol M200 silicone oil (density *d*_c_ = 0.970 cm^–3^, viscosity *ç*_c_ = 200 mPa s, loss factor tg *δ* = 0.0001, relative permittivity *ε´* = 2.89). The tested suspensions contained 5 wt.% of particles in silicone oil. The prepared suspensions were stirred by mechanical stirrer and then were sonicated for 30 s. The sedimentation stability was studied using a UV-vis spectrometer Varian (Varian, Palo Alto, CA, USA). 

Rheological measurements were carried out by means of Bohlin Gemini rotational viscometer (Malvern Instruments, Malvern, United Kingdom) under controlled-shear-rate (CSR) mode at 25 °C. Parallel plates with a diameter of 40 mm and a gap of 0.5 mm were used. 

#### 2.2.6. Methods for Composites Photo-Actuation Characterization

Samples in the form of the stripes with a length of 15 mm, width of 2.5 mm and thickness of 0.26 mm were cut from the prepared polymer composite (0.1 vol.% of neat GO or polymer modified GO particles in PDMS matrix), and were subjected to 10% pre-strain and exposed to irradiation of the red light emitting diode, LED, Luxeon Rebel (Philips, Amsterdam, Netherlands) for 10 s. The irradiation wavelength was 627 nm and source intensity – 6 mW light. The maximum value of actuation was characterized by changing the length of the sample during irradiation, Δ*L = (L_0_ − L)/L_0_*, where *L_0_* is the length of unexposed to the light sample mounted between 10 mm clamps and *L* is the length of the irradiated sample. Actuation describes a material’s ability to undergo reversible shape changes when subjected to an external light stimulus.

The thermal conductivity of the composite samples was performed on films, of the same specified shape and size, that were placed on the sensor and investigated using contact method and evaluated by the TCi model (C-term technologies, Fredericton, NB, Canada).

For optical images of the composites (Figure S1) a table microscope Leica (Leica, Tokyo, Japan) was used.

## 3. Results and Discussion

GO was prepared from the graphite by a modified Hummers method. The resulting synthesized particles were analyzed using XRD (Figure S2) and it was shown that there is just one peak at 17° indicating a well-exfoliated system. Further characterization was performed using AFM (Figure S3) to clearly show the morphology of this system and that there is only single layer sheet with 2 nm thickness and 2 μm width [46]. Surface area of GO was 9.04 m^2^ g^−1^.

**Scheme 1:**
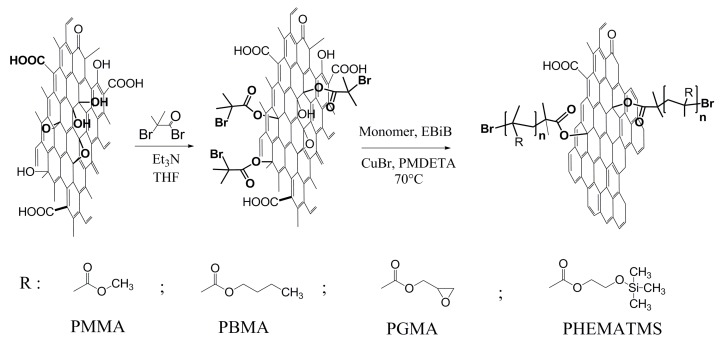
Schematic illustration of the modification of graphene oxide (GO) surface by atom transfer radical polymerization (ATRP) initiator and subsequent surface-initiated (SI-ATRP) of various monomers. R from the scheme represents corresponding monomeric moieties.

GO surface modification with PBMA, PMMA, PHEMATMS and PGMA was successfully performed using SI-ATRP. All polymerizations were stopped after two hours and then the formed GO-polymer hybrids were characterized. The characteristics of the polymer chains are summarized in Table 1. It can be seen that all materials have narrow *Đ* indicating good control over polymerization, while the monomer conversion was in the range of 41–67%. Thus, the degree of polymerization (DP) of the grafted polymers chains differed slightly. The DP was expected to increase in the following order: PBMA < PGMA < PMMA < PHEMATMS. Monomer conversion calculated from ^1^H NMR corresponded to the results from GPC. The grafted GO particles with various polymer moieties are summarized in Scheme 1, together with the scheme of initiator immobilization for subsequent polymer grafting.

**Figure 1:**
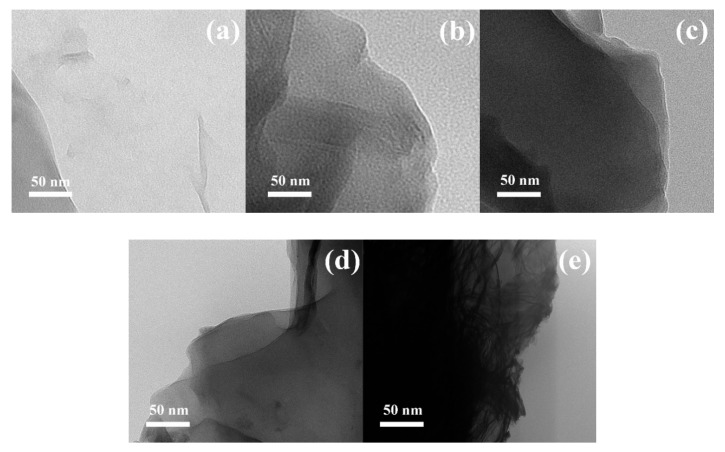
TEM images of: (**a**) the neat GO; and modifications with various polymers: (**b**) GO- poly(methyl methacrylate) (PMMA), (**c**) GO-poly(*n*-butyl methacrylate) (PBMA), (**d**) GO-poly(glycidyl methacrylate) (PGMA), (**e**) GO-poly(trimethylsilyloxyethyl methacrylate) (PHEMATMS).

TEM, TGA-FTIR, XPS and CA measurements were performed in order to directly confirm the success of grafting polymer chains from the GO surface. As can be seen in Figure 1, all samples, neat GO as well as modified analogues, possess a 2D shape. The proper exfoliation of neat GO particles (Figure 1a) can be seen and only several layers of individual GO sheets are present. After polymer modification, the contrast of the GO sheet is more pronounced, indicating the presence of a compact polymer layer (Figure 1b–e). For PBMA and PGMA moieties (Figure 1c,d), the contrast is even darker. This is most probably caused by several layers of modified GO sheets lying on top of each other. However, due to the fact that the edges of the GO sheets are smoother than the edges of neat GO, successful grafting can be determined. A similar situation is observed for GO-PHEMATMS (Figure 1e). In this case the DP and *M*_n_ of the coating are higher than for the rest of the samples and therefore, the contrast is the most pronounced. However, it also provides confirmation of a very compact layer and smooth edges. The neat GO was also investigated from the AFM point of view to show that we have nearly one layer GO with thickness up to 2 nm and width of several micrometers (Figure S3).

**Figure 2:**
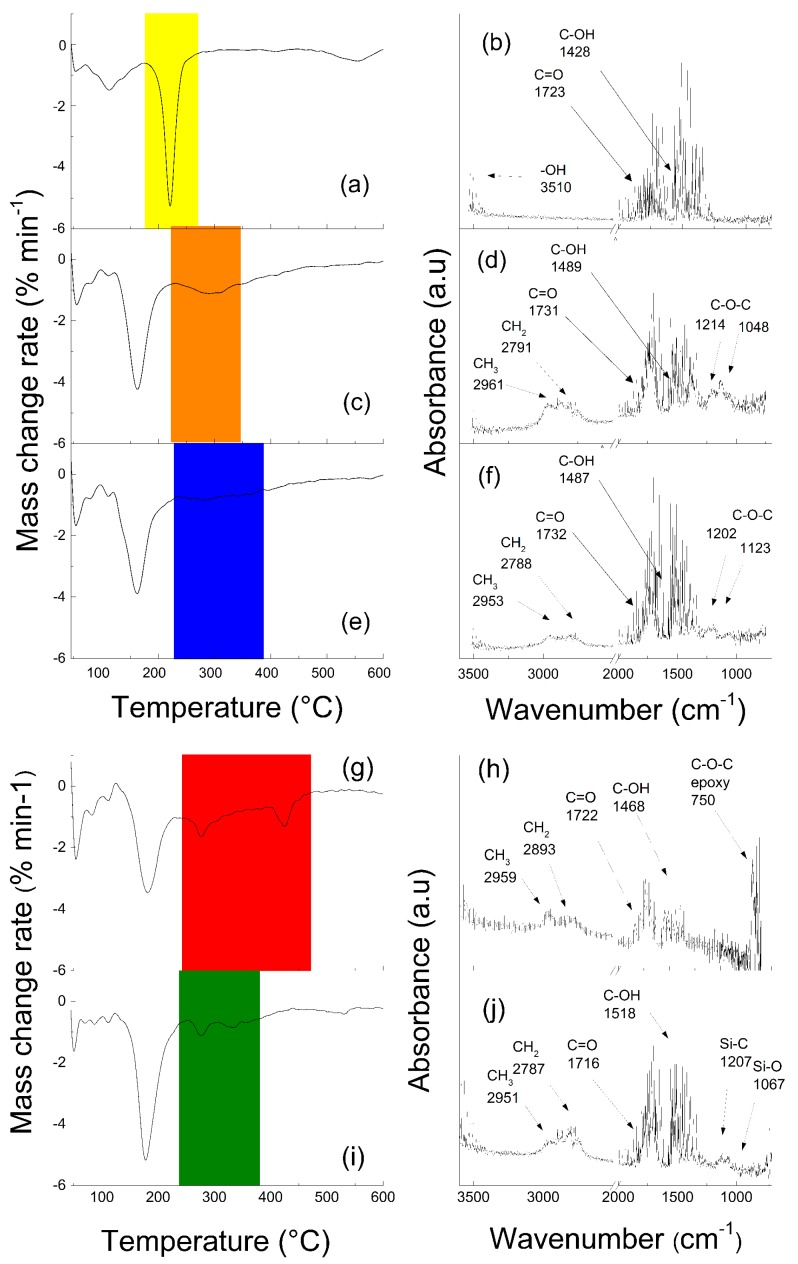
TGA-FTIR online monitoring of: (**a**,**b**) the neat GO, (**c**,**d**) GO-PMMA, (**e**,**f**) GO-PBMA, (**g**,**h**) GO-PGMA, (*i*,*j*) GO-PHEMATMS. Color strip corresponds to the decomposition of the oxygen containing groups (**a**) and individual polymer grafts (**b**–**e**)**.**

To confirm further the successful modification TGA, with coupled FTIR analysis, was performed to on-line monitor and evaluate the generated gas phase. The FTIR spectra shown in Figure 2b,d,f,h,j were collected in the temperature range of polymer decomposition, highlighted in colors. The neat GO (Figure 2a) had the highest amount of the oxygen-containing functional groups. Moreover, the position of the peak is at higher temperature indicating a relatively stable GO system [47]. The sharpness of the peak indicates the rather high thermal expansion of the neat GO system when high heating rates are used [47], while polymer modification slightly suppresses this effect. Moreover, in a case of polymer grafting from the GO surface, this peak is shifted to the lower temperatures, indicating that polymer bearing moieties allow the easier thermal decomposition of these functional groups, which is similar to results found by other authors [48]. Based on the FTIR it can be confirmed that -OH, -COOH as well as -C=O functional groups are present on the GO particles with absorption bands around 3500, 1428 and 1723 cm^−1^, respectively. For the GO hybrids modified by various polymer chains, the polymer decomposition is highlighted by color strip, while each color belongs to the specific moiety and sustains through the manuscript also for other characterizations. Here the absorption bands for methacrylate unit are the same for all polymers showing the absorption bands around 2950, 2800 and 1720 cm^−1^, for -CH_3_, -CH_2_ and -C=O, respectively. In a case of PGMA there is an additional significant peak at about 750 cm^−1^ due to the presence of the epoxy ring in the polymer structure. PHEMATMS differs owing to the presence of the Si-O and Si-C bands at 1047 cm^−1^ and 1201 cm^−1^. It can also be seen in Table 2, that the density of the particles changed slightly after SI-ATRP indicating the presence of polymer layer at the GO surface.

**Figure 3:**
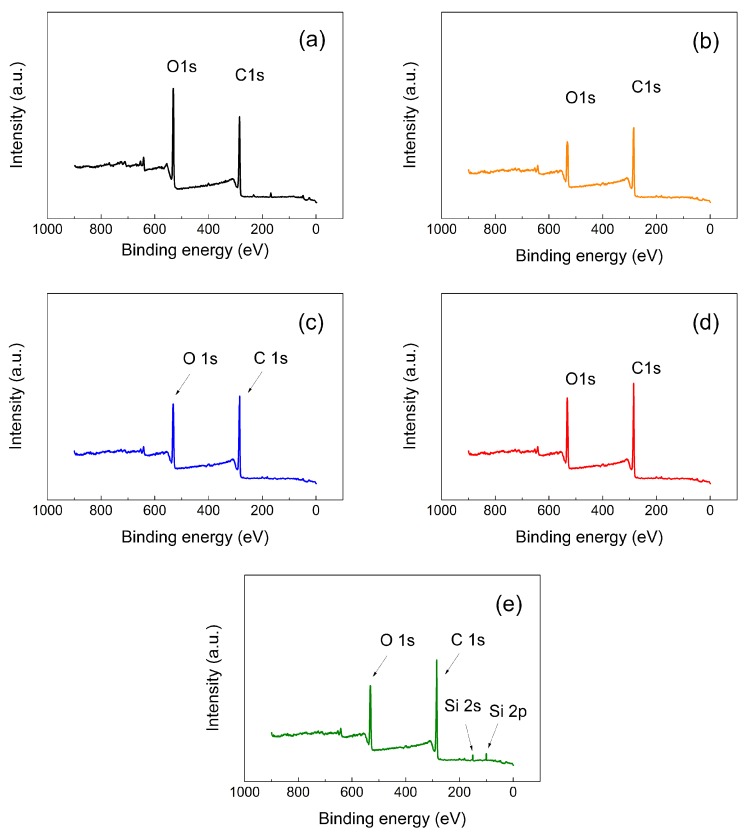
XPS spectra of: (**a**) the neat GO, (**b**) GO-PMMA, (**c**) GO-PBMA, (**d**) GO-PGMA, (**e**) GO-PHEMATMS.

The XPS spectra of neat GO (Figure 3a) also confirmed that functionalization during oxidation was successful because a significant amount of oxygen was detected. The oxygen containing groups in neat GO consisted of 54% C-O, 22% C=O and 24% of COOH. The ratio between C1s and O1s was changed after the grafting of GO with PMMA chains, due to the presence of the polymer as well as partial reduction of accessible GO surface. A similar trend was observed for the GO-PBMA and GO-PGMA, showing the increased C/O ratio. The successful modification with PHEMATMS was further confirmed by the appearance of new absorption bands from Si 2s and Si 2p, showing the covalent bonding of the polymer layer. The atomic content of all investigated samples is shown in Table 2.

**Figure 4:**
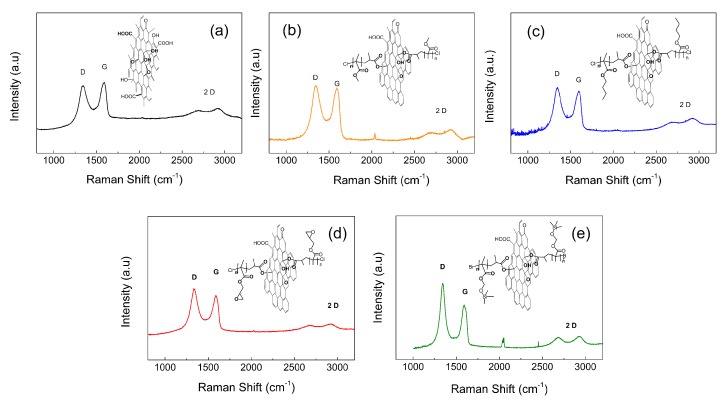
Raman spectra of: (**a**) the neat GO, (**b**) GO-PMMA, (**c**) GO-PBMA, (**d**) GO-PGMA, (**e**) GO-PHEMATMS.

GO particle reduction during SI-ATRP modification was confirmed by two independent methods; conductivity investigations (Table 2) and Raman spectroscopy (Figure 4). Both techniques showed that the reduction of GO took place and the conductivity was finely tuned by the structure of the polymer grafts. The D and G peaks intensities were elucidated to properly investigate the GO reduction and quantify the degree of reduction. The increase *I*_D_/*I*_G_ from 0.9 to 1.05, 1.08, 1.08 and 1.09 for neat GO, GO-PMMA, GO-PBMA, GO-PGMA and GO-PHEMATMS, respectively, was observed. The slight reduction during the SI-ATRP is caused by the presence of the excess of a tertiary amine, commonly used as a ligand complexing the copper catalyst, as was already observed and described previously by our group [37]. The reduction usually depends on the reaction conditions such as polymerization time, tertiary amine concentration and tertiary amine to GO and tertiary amine to monomer ratio. Due to the same polymerization conditions for all graftings reported in this study, the highest conductivity for GO-PHEMATMS can be attributed to the highest polarizability of the pendant groups.

**Figure 5:**
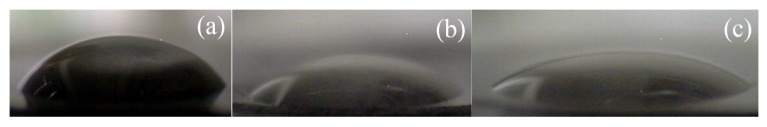

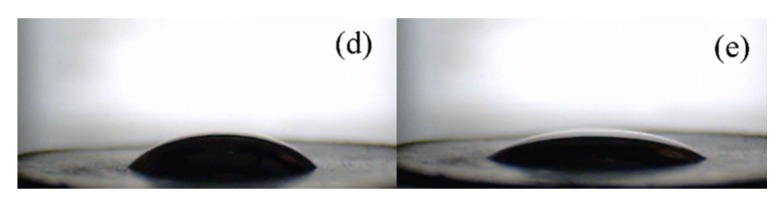
Contact angle investigations using sessile drop method on: (**a**) the neat GO, (**b**) GO-PMMA, (**c**) GO-PBMA, (**d**) GO-PGMA, (**e**) GO-PHEMATMS.

**Figure 6:**
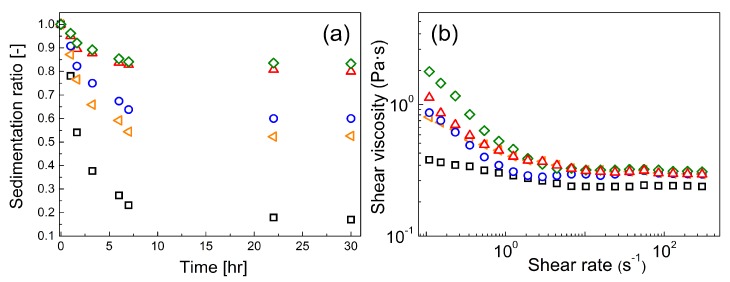
(**a**) Sedimentation ratio and (**b**) off-state viscosity in the absence of the external electric field for: (□) neat GO, (◁) GO-PMMA, (○) GO-PBMA, (△) GO-PGMA, (◊) GO-PHEMATMS.

The compatibility of the neat GO and polymer-grafted GOs with the silicone-based environment was investigated by contact angle measurements (Figure 5), determination of the sedimentation ratio (Figure 6a) and the steady shear viscosity profiles (Figure 6b). As shown in Figure 5, the contact angle of neat GO and GO hybrids decreased from 49.9° ± 3.2° to 38.7° ± 2.7°, 28.7° ± 2.7°, 30.1° ± 1.7°and 26.3° ± 3.0° for neat GO, GO-PMMA, GO-PBMA, GO-PGMA and GO-PHEMATMS, respectively. These results confirmed the enhanced ability of the system to be compatible with the silicone-based environment.

It can be observed from the sedimentation stability profiles that the GO-PHEMATMS system exhibited significantly enhanced stability, which also correlates with steady shear investigations where the off-state viscosity is also the highest. The results followed the trend from the CA measurements and confirmed that the compatibility of the hybrid particles with the environment could be modulated over a wide range by the polymer graft structure. Thus, tailoring of the properties of the system could be achieved. 

**Figure 7:**
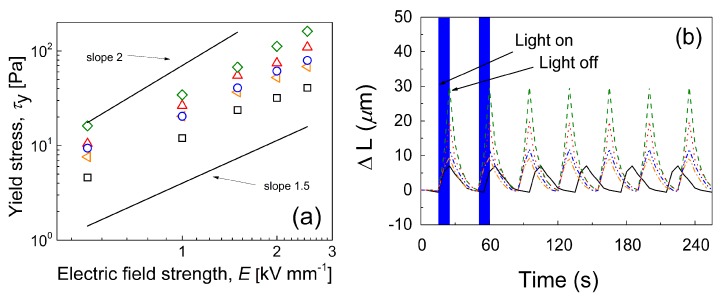
(**a**) Yield stress dependence on the electric field strength and (**b**) photoactuation capability by means of sample contraction, where: neat GO is (□) and solid black line, GO-PMMA is (◁) and orange dash-dot line, GO-PBMA is (○) and blue dash-dot-dot line, GO-PGMA is (△) and red dotted line and GO-PHEMATMS is (◊) and green dashed line.

Finally, electrorheological (Figure 7a) and photo-actuation (Figure 7b) investigations were performed, in order to prove the impact of the different moieties on the capability of the stimuli-responsive properties. In the first case, the yield stress, *τ*_y_, as a measure of the rigidity of the internal chain-like structures, was plotted versus external electric field strength, *E*. The power-law model *τ*_y_ = *q E^m^* was used for investigation of the ER performance, where *q* corresponds to stiffness of the internal chain-like structures and the exponent (*m)* reaches values 1.5 for conductivity mechanism (medium and particles conductivity mismatch is responsible for chain-like structure development) and reaches 2 for the polarization mechanism (medium and particles relative permittivity mismatch is accountable for ER behavior). All systems exhibited the conductivity mechanism [49,50] of the chain-like structure formation (Figure 7a) since the dependence for all samples essentially followed the slope of 1.5 (Table 3) rather than 2.0, as was observed elsewhere [37]. Moreover, based on the results from conductivity (Table 2) the ER performance is mainly depended on the conductivity mismatch between silicone oil and the particles. Therefore, the highest deviation from the ideal behavior was found for neat GO exhibiting the yield stress just of 40 Pa at 2.5 kV mm^−1^, while the GO-PHEMATMS system shows ideal behavior and has the best ER performance with yield stress of 200 Pa at 2.5 kV mm^−1^. 

In the case of a photo-actuation investigation, the Δ*L* was used as the contraction length after irradiation. Here the contraction ability is mainly affected by the stiffness of the polymer matrix and proper redistribution of the heat within the sample, which is ensured by appropriate particle dispersion. In this case, the better heat redistribution also is associated with higher electric conductivity of samples, where the GO-PHEMATMS benefits. It has to be noted, that the slower and in fact delayed response of the neat GO-based composite to the light stimulus is connected to the abovementioned reasons such as improper particle distribution, as well as low electrical properties (Table 2). Therefore the determined thermal conductivity for neat GO, GO-PMMA, GO-PBMA, GO-PGMA and GO-PHEMATMS was equal to 0.134, 0.153, 0.155, 0.161 and 0.167 W m^−1^ K^−1^, respectively. Due to these facts the heat redistribution in the sample is more complicated and transformation from light to mechanical energy needs more time. As was also investigated by our group, the short polymer chains act as plasticizers, and even if they are grafted from the reinforcing surface like carbon nanotubes or carbonyl iron, the mechanical properties are lower [10,15,51]. In good agreement with the literature, all types of polymer chains grafted from GO provided a plasticizing effect, therefore the photo-actuation performance was better in comparison to the neat GO. The best properties could be seen for GO-PHEMATMS, which also exhibited the best compatibility with the silicone-based environment and thus provided the system with the best particle dispersibility and the best heat redistribution. This sample showed nearly 30 μm contraction after 10 s of irradiation, which is enormously high in comparison to other GO hybrids and/or previously investigated PDMS based systems [9,25]. Unfortunately, as already mentioned 30 μm of actuation for our best chemically cross-linked PDMS systems are not that high in comparison to those GO-hybrids dispersed in the TPEs, due to the fact the physical cross-linking provides better flexibility of the systems, however this is not that stable from the long-term on/off cycling point of view [52,53].

## 4. Conclusions

This study discusses the approach of modification of the GO surfaces by various polymethacrylates with simultaneous partial reduction of the GO to the degree applicable in electrorheology systems. The various polymers such as PMMA, PBMA, PGMA and PHEMATMS were grafted from GO and characterized. The GO reduction was confirmed by Raman spectroscopy as well as conductivity measurements showing that GO-PHEMATS provided a material with finely tuned conductivity. This was due to the involvement of the effect of the dipole moment of the pendant groups of the grafted polymer chains. The effect of compatibility of the fillers with the silicon-base environment was investigated using three separate approaches; contact angle, sedimentation stability and steady shear viscosity investigation, and all methods provided the same trend confirming the best compatibility of GO-PHEMATMS thanks to the similarity of its structure to the silicone-based environment. Finally, since the GO-PHEMATMS possessed the best electric properties, it also provided the best ER performance, where the yield stress increasing in the row neat GO < PMMA < PBMA < PGMA < PHEMATMS, showing values 40, 68, 79, 110 and 200 Pa, respectively. Such results are very promising for potential application especially for such low particles loading. Furthermore, thanks to the significantly improved particles compatibility their improved dispersibility in PDMS could be expected, and together with considerable plasticizing effect and improved heat redistribution the GO-PHEMATMS also provided a system with enhanced contraction length upon irradiation in the same row as in electrorheology, showing contraction nearly of 7, 9, 11, 19 and 30 μm, respectively, which is very promising value and was not observed on PDMS-based systems under similar testing conditions. Our studies confirmed the importance of tailoring the nature of the particles surface to obtain the best compatibility with the continuous phase and thus to achieve desired final properties of the material for applications in electrorheology or as photo-mechanical actuators.

## Figures and Tables

**Table 1:** Molar ratios of individual reactants and characterization of polymers grown form sacrificial initiator.

Sample Name	M ^a^	I ^a^	L ^a^	CuBr	*M*_n_^b^ (g mol^−1^)	*Đ* ^b^	Conversion ^c^ (%)
GO-PMMA	100	1	4	1	5620	1.18	59
GO-PBMA	100	1	4	1	5210	1.21	41
GO-PGMA	100	1	4	1	5920	1.23	53
GO-PHEMATMS	100	1	4	1	12,600	1.19	67

^a^ M, I, L stays for monomer, sacrificial initiator and ligand; ^b^ according to the GPC; ^c^ according to the ^1^H NMR.

**Table 2:** Densities, surface elements content and conductivities of neat GO and GO-modified particles.

Sample Name	Density (g cm^−3^)	Surface Element Content ^a^				Conductivity (S cm^−1^)
		C	O	Si	C/O	
neat GO	2.68	66.7	33.3	0	2.00	1.2 × 10^−8^
GO-I	2.64	67.2	32.8	0	2.05	1.9 × 10^−8^
GO-PMMA	2.53	69.1	30.9	0	2.24	6.3 × 10^−8^
GO-PBMA	2.34	70.9	29.1	0	2.43	2.1 × 10^−7^
GO-PGMA	2.28	72.3	27.7	0	2.61	3.0 × 10^−7^
GO-PHEMATMS	2.39	73.1	24.9	2.0	2.94	6.0 × 10^−6^

^a^ based on XPS.

**Table 3:** Parameters of power-law model fits of neat and polymer modified GO particles suspensions.

Sample Code	q [Pa]	m
GO	7.6	1.47
GO-PMMA	20.8	1.48
GO-PBMA	24.6	1.48
GO-PGMA	28.7	1.49
GO-PHEMATMS	41.6	1.5

## Supplementary Materials

The following are available online at https://www.mdpi.com/2079-4991/10/3/591/s1, Figure S1: Optical images of the prepared PDMS composites containing 0.1 vol.% of neat GO (a), GO-PMMA (b), GO-PBMA(c), GO-PGMA (d) and GO-PHEMATMS (e), Figure S2: XRD pattern for graphite and corresponding neat GO, Figure S3: AFM image of neat GO, Figure S4: TGA spectra of the neat GO and GO hybrid particles. Color strip corresponds to the color strip in the manuscript and reflecting the decomposition of the oxygen containing groups (Figure S4a) and individual polymer grafts (Figure S4b–S4e).
