# Supplementary material for: Effect of Structure of Polymers Grafted from Graphene Oxide on the Compatibility of Particles with a Silicone-Based Environment and the Stimuli-Responsive Capabilities of Their Composites
Source: Nanomaterials (Basel). 2020 Mar 24;10(3):591. doi: 10.3390/nano10030591 (PMC7153385; doi:10.3390/nano10030591)
Supplement: Supplementary file 1 [file nanomaterials-10-00591-s001.pdf]

Effect of Structure of Polymers Grafted from Graphene Oxide on the Compatibility of Particles with a Silicone-Based Environment and the Stimuli-Responsive Capabilities of Their Composites

Monika Zygo [1], Miroslav Mrlik [2,*], Marketa Ilcikova [1,3], Martina Hrabalikova [2], Josef Osicka [2], Martin Cvek [2], Michal Sedlacik [2], Barbora Hanulikova [2], Lukas Munster [2], David Skoda [2], Pavel Urbánek [2], Joanna Pietrasik [1,*], Jaroslav Mosnáček [3,4,5,*]

[1] Department of Chemistry, Lodz University of Technology, Institute of Polymer and Dye Technology, Stefanowskiego 12/16, 90 924, Lodz, Poland
[2] Centre of Polymer Systems, University Institute, Tomas Bata University in Zlin, Trida T. Bati 5678, 760 01 Zlin, Czech Republic
[3] Polymer Institute, Slovak Academy of Sciences, Dubravska cesta 9, 845 41 Bratislava 45, Slovakia
[4] Department of Polymer Engineering, Faculty of Technology, Tomas Bata University in Zlin, Vavreckova 275, CZ-76272 Zlin, Czech Republic
[5] Centre for Advanced Material Application, Slovak Academy of Sciences, Dubravska cesta 9, 845 11 Bratislava, Slovakia
* Correspondence: mrlik@utb.cz (M.M.), joanna.pietrasik@p.lodz.pl (J.P.), jaroslav.mosnacek@savba.sk (J.M.)

SUPPORTING INFORMATION

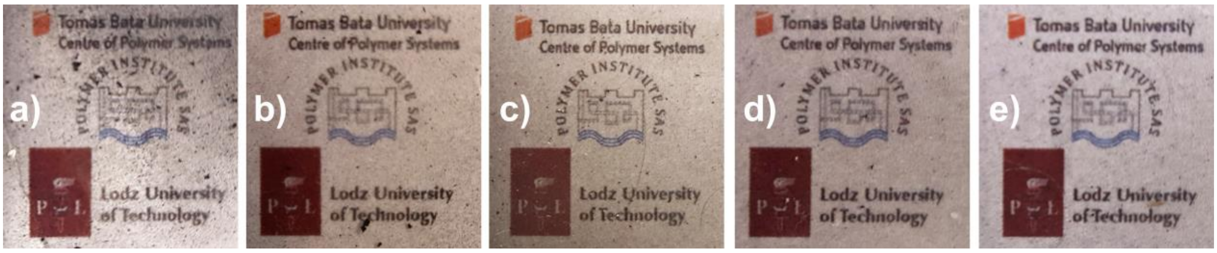

Fig. S1: Optical images of the prepared PDMS composites containing 0.1 vol.% of neat GO (a), GO-PMMA (b), GO-PBMA(c), GO-PGMA(d) and GO-PHEMATMS (e).

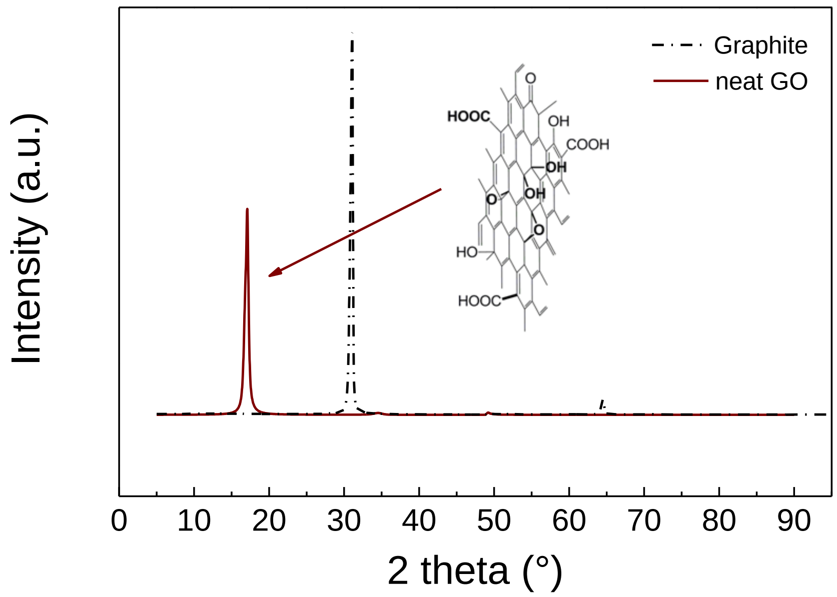

Fig. S2: XRD pattern for graphite and corresponding neat GO.

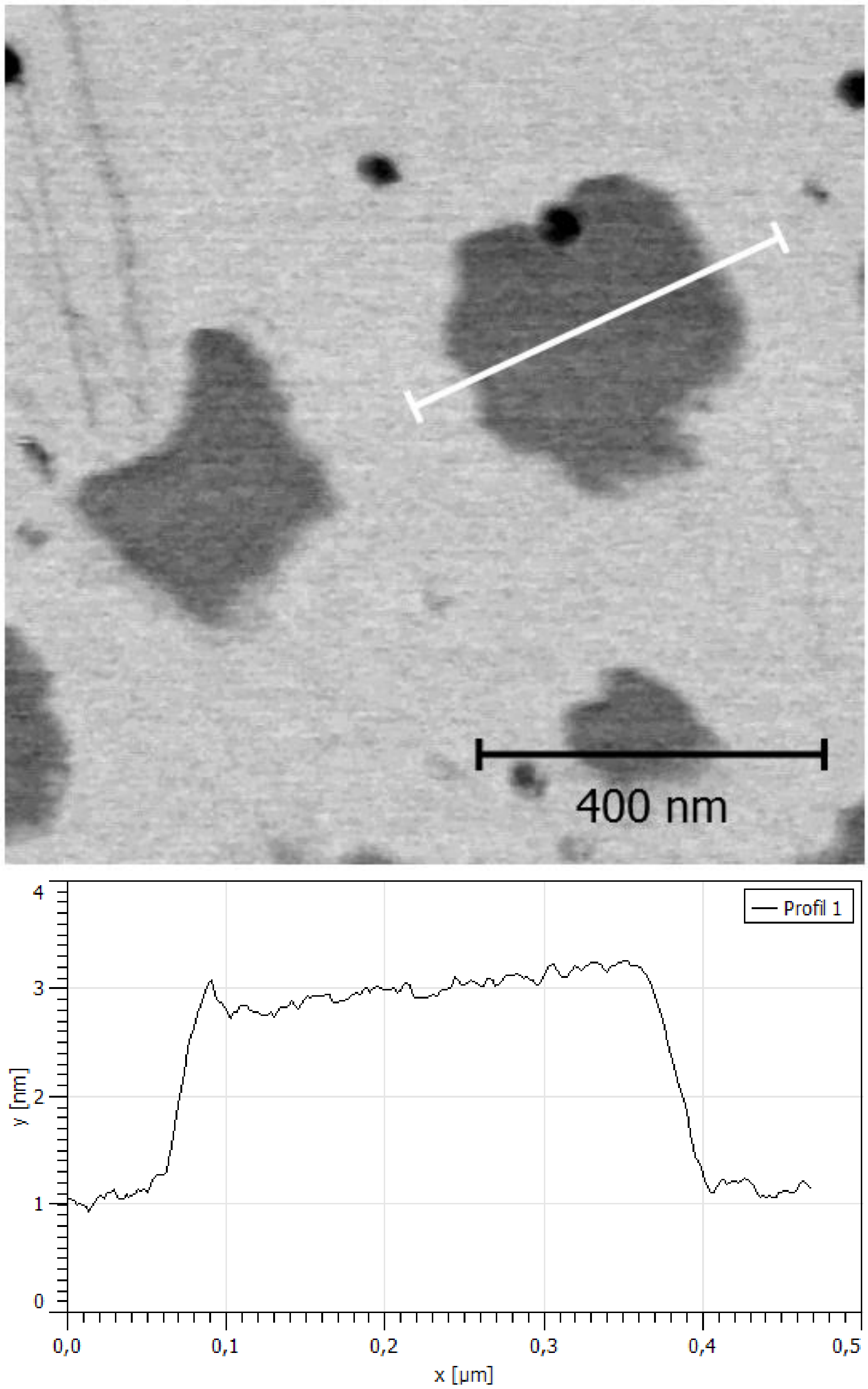

Fig. S3: AFM image of neat GO.

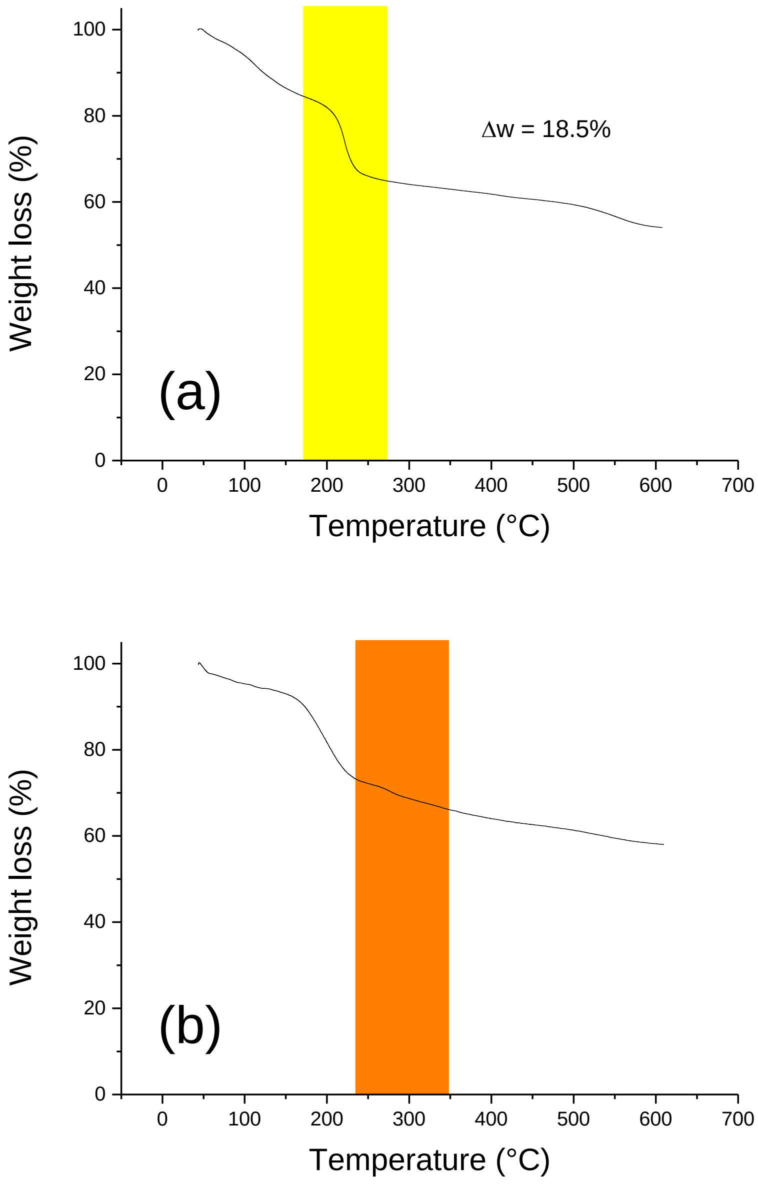

Weight loss (%)
Temperature (°C)
Δw = 18.5%
(a)
(b)
0
20
40
60
80
100
0
100
200
300
400
500
600
700

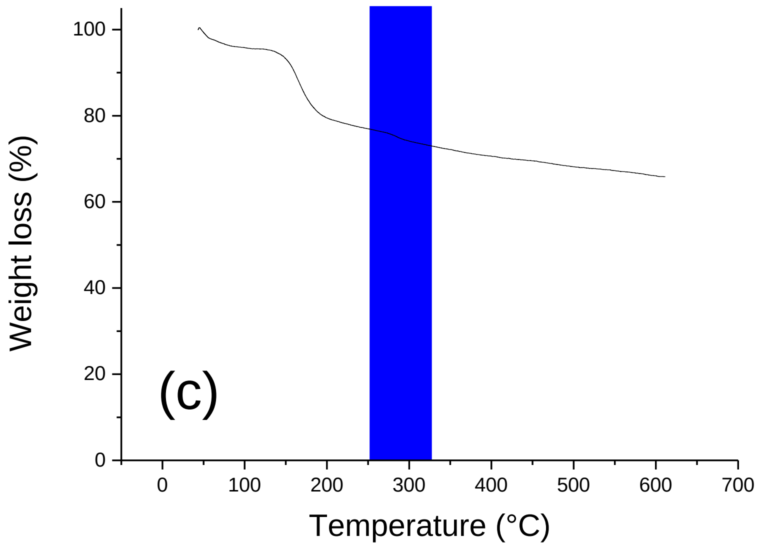
100
80
60
40
20
0
Weight loss (%)
(c)
0
100
200
300
400
500
600
700
Temperature (°C)

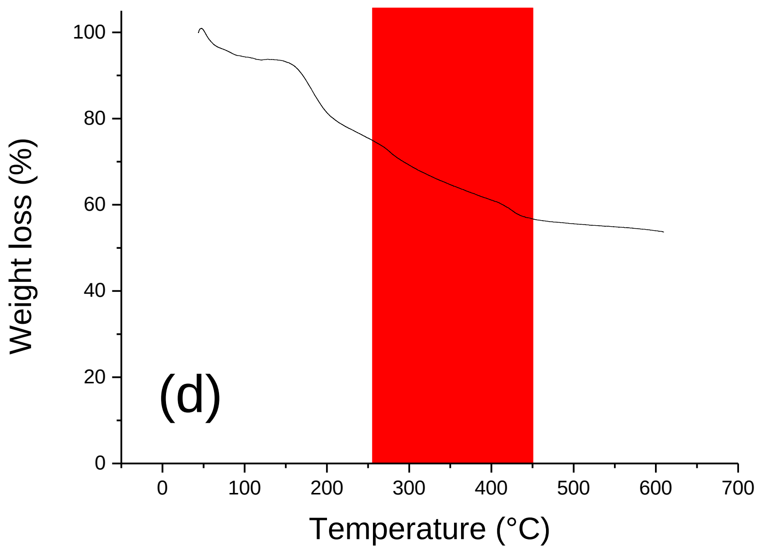
100
80
60
40
20
0
Weight loss (%)
(d)
0
100
200
300
400
500
600
700
Temperature (°C)

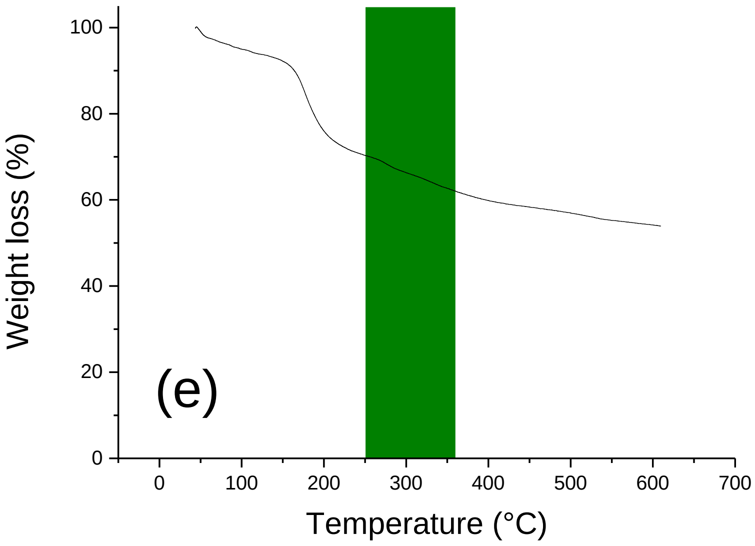

Fig. S4: TGA spectra of the neat GO and GO hybrid particles. Color strip corresponds to the color strip in the manuscript and reflecting the decomposition of the oxygen containing groups (Fig. S4a) and individual polymer grafts (Figs. S4b-S4e).
